# Criptiquing Disability Models: Compulsory Able‐Bodiedness, Nursing Practice, and Reimagined Disability

**DOI:** 10.1111/nup.70075

**Published:** 2026-03-01

**Authors:** Teresa A. Graziano

**Affiliations:** ^1^ Department of Nursing University of Vermont Burlington Vermont USA

**Keywords:** crip nursing, crip theory, disability justice, nursing philosophy

## Abstract

Within nursing education and practice, disability has been shaped by models of disability that narrowly focus on rehabilitating the body to a normative, able‐bodied state. This has profound moral implications for nursing practice and affects the nurse‐patient dynamic. Typically, this empowers the nurse while disempowering the disabled person by portraying the nurse as the expert in disability and the disabled person as a passive recipient of care. This paper applies crip theory, specifically McRuer's framework of compulsory able‐bodiedness, to critically examine the enduring presence of the charity, moral, and medical models of disability in nursing education and practice. Compulsory able‐bodiedness is the system that produces disability by othering non‐normative bodies. This produces a hierarchy of bodily normalcy, with people who are able‐bodied at the top, people with visible disabilities in the middle, and people with invisible disabilities at the bottom. I argue that these three historically dominant models are etic, ignore disabled people's voices, and perpetuate ableist assumptions that limit the profession's capacity to support disabled people in liberatory and socially just ways. I then present an alternate crip paradigm: the social model of disability. The social model of disability offers nurses the opportunity to honour the disabled person as the expert in their care, focus on the individual's strengths rather than deficits, and recognise the complex interplay between physiological, social, political, and environmental factors that contribute to the experience of disability. In raising this critical consciousness, I call for a reimagined nursing education and practice that honours disability as a source of knowledge, transformation, and resistance.

1

Disabled people, particularly in healthcare settings, are simultaneously hypervisible and invisible, often feeling ‘stared at and unseen’ (Lakshmi‐Piepzna‐Samarasinha 2022, p. 160). Disability has long been framed within healthcare education and practice through myopic paradigms (hereafter ‘models of disability’) that emphasise cure, correction, and the return of the body to a normative, able‐bodied standard (Scullion 2010; McMillan Boyle et al. 2008). Nursing education and practice are shaped by the moral, charity, and medical models of disability, which are limited by ableist assumptions and barriers. These three interrelated models position disabled people as tragic burdens and deficient sufferers in need of fixing. As I will outline, they not only inform pedagogical approaches and clinical decision‐making but also perpetuate ableist assumptions that constrain how nurses engage with disabled people in real‐world settings.

As a disclaimer, ‘cripple’ was historically used to derogatorily describe physically disabled people. As the disability rights movement challenged ableism in American society through the late 20th century, ‘cripple’ and ‘crip’ were reclaimed by many disabled people as a term of empowerment and solidarity (Schalk 2013; Schoenherr‐Wustl 2019) and in rejection of patronising language like ‘differently abled’ (Lewis 2015). Identifying as crip is not limited to those with physical or severe intellectual disabilities, which may be referred to as visible disabilities that are readily apparent to others. Crips may be invisibly disabled, such as the ‘mad’, or people with psychological conditions (e.g., major depressive disorder, schizophrenia), Deaf (who may identify as language minorities, rather than disabled), neurodivergent individuals, individuals with chronic illnesses (e.g., fibromyalgia, diabetes, etc.), and those who otherwise deviate from a normative state of mind or body (Lakshmi Piepnza‐Samarasinha 2022; Lakshmi Piepzna‐Samarasinha 2018; Americans With Disabilities Act of 1990 1990). When referring to disabled people throughout this paper, I am referring to people with both visible and invisible disabilities who may be diagnosed or undiagnosed. Also, in line with crip practice, I use identity‐first language throughout this paper, rather than person‐first language (i.e., ‘disabled person’ vs. ‘person with disabilities’), as most disabled people prefer the former (Brown 2023).

In this paper, I compare the moral, charity, and medical models of disability and contrast them with a fourth model, known as the social model. Broadly, the moral model presents disability as a sign of sin, weakness, or personal failing. The charity model presents disabled people as objects of pity who require benevolence from able‐bodied people. The medical model frames disability as a defect of the body that must be cured, rehabilitated, and erased. Other nurse scholars have made the case against the moral, charity, and medical models of disability, endorsing the social model (Scullion 2010; Boyles et al. 2008). The purpose of this paper is to critique (hereafter ‘criptique’, to align with critical disability studies; See Wood 2014) these three traditional disability models in nursing using McRuer (2006) crip theory of compulsory able‐bodiedness. I will then reintroduce the social model, which is not driven by the same assumptions as traditional models of disability, to demonstrate how models of disability continue to influence nursing practice and education. This will culminate in a reimagining of nursing education and practice that embraces disability as a source of knowledge, resistance, and transformation.

## 2 Crip Theory and Compulsory Able‐Bodiedness

Crip theory is the term used to describe the philosophical intersection of queer theory and critical disability studies, which considers the intersections of race, gender, class, and other axes of identity with disability (McRuer 2006; Sins Invalid 2015; Schalk 2013). Crip theorists link queerness with disability, as both identities (i.e., queer and disabled) share a history of pathologization rooted in their variation from normative embodiments of sexuality, gender, and ability (McRuer 2006). Queer theorists partially posit how heteronormativity, or the belief that the human default is heterosexuality, harms individuals because any sexuality that deviates from the [heterosexual] norm is pathologized, and the mind‐bodies that have sexual responses to same‐sex partners are subsequently deemed faulty. This has led to historical attempts to ‘cure’ one's homosexuality through various interventions and therapies (Graham 2019), an act that nurses were/are complicit in (Dickinson 2014). Critical disability theorists similarly question how ableism, or the stigmatisation associated with being disabled in a society that values able‐bodiedness, pathologizes differences and nonconformity in the body (Lakshmi Piepzna‐Samarasinha 2018; McRuer 2006). This pathologization of disability is then used to justify the disempowerment and marginalisation of individuals who do not fit normative expectations. Crip theory examines heteronormativity and ableism together to critically appraise the shared assumptions that produce stigmatisation of non‐normative bodies as ‘deviant’ or ‘other’.

For this analysis, nursing will be situated within McRuer's crip theory of compulsory able‐bodiedness (2006). Compulsory able‐bodiedness is the system that produces disability by othering non‐normative bodies and minds (McRuer 2006, p. 2). In prioritising the able‐bodied and ‐minded, able‐bodiedness ‘largely masquerades as a non‐identity, as the natural order of things’ (p. 1). Compulsory able‐bodiedness assumes the existence of an achievable, perfect, nondisabled state which harms the disabled and able‐bodied alike, given that ‘everyone is virtually disabled… able‐bodied status is always temporary, disability being the one identity category that all people will embody if they live long enough’ (McRuer 2006, p. 30). Able‐bodiedness is fragile, and as chronic conditions develop or unexpected accidents occur, it slips through one's fingers and rehabilitation is not always achievable.

## 3 Criptiquing the Traditional Models of Disability in Nursing

In this section, each model of disability will be criptiqued to demonstrate how each etic model pathologizes and disempowers disabled people. Table 1 presents a brief summary of the three traditional model of disability, as well as the crip social model. Criptiquing the models of disability utilised by nurses is increasingly necessary. Nurses care for people who are disabled either temporarily by an acute ailment or permanently from various complex causes. Even people seeking nursing care who are not disabled today will become so someday, as the human body inevitably ages and becomes increasingly dependent on others for care (Chochinov 2023). The care given to a patient is predicated on the nurse's mindset and beliefs about disability, or model of disability. Moral, charity, and medical models of disability are embedded even before nursing education through existence in an ableist society that views disabled bodies as degraded versions of an abled body. Nurses are predominantly able‐bodied or deny their own disabilities to do the demanding work of a nurse (Neal‐Boylan and Miller 2020; Valdez 2024). Because of societal manifestations of the moral, charity, and medical models of disability, many nurses are primed to think of disabled people as passive recipients of care who uniformly have a poor quality of life, and whose dignity the nurse must grant through care. Through socialisation under a system of compulsory able‐bodiedness, nurses are conditioned to perceive disabled individuals as either ‘supercrips’ who are disabled people who overcome their disability to achieve radical independence or ‘pathetic cripples’ who are dependent on others for the most basic care (Lakshmi Piepzna‐Samarasinha 2022, p. 156; Lakshmi Piepzna‐Samarasinha 2018, p. 35). On either end of the binary, the disabled person is rarely viewed as human outside of the context of their disability (Lakshmi Piepzna‐Samarasinha 2022), and this negatively impacts the people nurses care for (Valdez 2024). By using crip theory to examine the assumptions of the moral, charity, and medical models, nurses can transform their pedagogical and practice approaches to better reflect the needs of people disabled by acute and chronic illnesses, represented in the social model.

**TABLE 1:** A comparative overview of four models of disability.

	Moral model	Charity model	Medical model	Social model
Core Belief	Disability is the result of sin, moral failing, or spiritual lack	Disabled people are tragic victims deserving of help and compassion from able‐bodied people	Disability is a physical or mental impairment that must be treated or cured	Disability arises from societal barriers, not the impairment itself
View of the Disabled Person	Object of shame or punishment, often hidden/pitied	Passive recipients of kindness, dependent and helpless	Patient who needs diagnosis, treatment, and/or rehabilitation to able‐bodied state	Rights‐holder, oppressed by inaccessible environments
Source of the Disability	Disability comes from an individual's moral failing, and/or is a test of their faith	Disability is a personal tragedy that has happened to them	Physiological or psychological dysfunction or abnormality	Disability is a social construct rooted in inaccessibility
Common Language/Imagery	Burden, curse, deserved suffering	Inspiring, brave, heartwarming	Suffering from, wheelchair‐bound, normal versus abnormal	Disabled by society, barrier‐free, inclusive
Goal of Intervention	Spiritual and divine acceptance	Rescue the person from their suffering, and generate sympathy from the public	Cure the body to be free of disability	Political, economic, social, and policy systems that have increased access and inclusion
Implications for Practice	Focus on atonement and spiritual interventions	Reinforces power imbalances	Emphasises the need for normalisation via rehabilitation and cure	Rejects pathologization of disability
	A person feels a need to conceal their disability	Supports paternalistic care	Promotes benevolence and charity	Focus on accessibility, universal design, and systemic change
	Stigma persists in care settings	Service for but not by disabled people	Service for but not by disabled people	Promotes the agency of the individual as the expert in their disability
		Promotes dependency narratives	Disability is seen as an individual burden	Justice‐oriented

### 3.1 Criptique of the Moral Model

The moral model of disability, which judges disability to be a personal failing, sin, or consequence of flawed character, has long shaped cultural narratives around disabled bodies (Olkin 2002), and is used by most world religions. Within nursing, this model may not always be overt, yet it persists in subtle forms of moral judgement embedded in language, practice, and education. Nurses may, for instance, attribute a disabled person's health status to lifestyle choice or lack of personal responsibility, echoing the moral model's logic that disability results from individual failings rather than a complex interaction between physical, political, social, and structural environmental conditions. A typical example of the moral model in contemporary nursing practice is the continued use of the term noncompliance and its euphemisms, nonadherence and nonconcordance. When patients are designated noncompliant, they're perceived as ‘bad’ patients who do not follow the nurse's rehabilitative instructions (Russell et al. 2003).

Compulsory able‐bodiedness depends on an unspoken hierarchy of bodily normalcy. Because of ableist dogma, visible and invisible disabilities are tolerated or stigmatised differently. While disability is stigmatised at baseline, there is a socially‐enforced hierarchy of stigma in which those with visible disabilities receive the least amount of stigma because onlookers can readily identify them as disabled, and those with invisible disabilities receive the most stigma because they do not necessarily embody ableist assumptions of what a disabled person looks like (Smart 2019). This hierarchy of stigma is inversely correlated with accessible services, such that those with visible disabilities have greater and easier access to services than those with invisible disabilities, who undergo more scrutiny when requesting similar resources, as they may not embody stereotypes of a disabled person (Smart 2019). For example, when a person with an invisible disability like fibromyalgia uses a handicap parking spot and walks into a store unassisted, onlookers may consider this person to be untruthful or abusing a system designed for someone who looks disabled, like a wheelchair user or someone who uses assistive devices. This results in the person with invisible disability experiencing greater stigma, as they may be accosted by onlookers who demand that the disabled person perform able‐bodied notions of disability or disclose health conditions to justify the use of the handicapped parking space.

This hierarchy of bodily normalcy, rooted in the moral model, classifies certain bodies as proper and judges others as deviant, irresponsible, or wrong. The moral model of disability functions to maintain this hierarchy by assigning blame to those who do not meet able‐bodied ideals, or those who do not want to, and in doing so, naturalises disability as a moral flaw rather than interrogating systemic conditions that shape health and impairment. For example, when thinking about a patient deemed noncompliant under the moral model, it is easy to conclude that the noncompliant patient simply does not want to get better (i.e., return to able‐bodiedness), and therefore the patient is undeserving of extra effort on the caregiver's side; ‘the patient isn't trying to get better, so why should I help them?’ This logic results in a hierarchy of who is deemed deserving of nursing care (i.e., those who are perceived as wanting to get ‘better’ [able‐bodied]) and those who are not (i.e., those who are not perceived as wanting to get ‘better’ [able‐bodied]). Ultimately, this results in nurses caring differently for patients depending on how likely the person receiving care is to follow the nurse's instructions to return to achieve able‐bodied ideals of health, regardless of patient autonomy and external barriers.

By placing blame for a disability on the individual, the moral model encourages nurses to presume personal deficiency rather than challenging broader social and institutional barriers. It reinforces an individualistic logic of personal virtue and self‐reliance, thus pushing the person away rather than emphasising interdependence, where a nurse can be a care resource. In practice, this manifests as a lack of empathy or curiosity about disabled people's lived experiences and needs since their difference is predetermined to be a kind of moral failure. This is exacerbated when a patient is ‘bad’ and does not try to, or cannot, rehabilitate. This contradicts nursing's value of holistic care. By rejecting the moral model, nurses can shift focus from personal blame to collective responsibility for dismantling barriers. In doing so, nurses can reimagine their roles as partners in resisting ableism and affirm the inherent worth of all bodies which better aligns with a nursing model of care.

### 3.2 Criptique of the Charity Model

The charity model of disability frames disabled people as passive objects of pity who depend on the benevolence of able‐bodied people for survival and support (Ralph 2017). Within nursing, this continues to shape subtle and explicit attitudes, casting nurses as rescuers or saviours and disabled people as grateful recipients of care. This implies nurses provide the care they believe the disabled person needs and wants, and always do so with the patient's interest in mind. A nurse practicing within the charity model of disability might be seen speaking on behalf of a patient who has difficulty with speech but can otherwise communicate, assume disabled patients cannot understand or make informed decisions for themselves, frames their care as doing a good deed or being kind to someone less fortunate, and prioritising comfort out of sympathy rather than empowering the patient to be an active member in their care and decision making process. This again disempowers the patient who is perceived as dependent and passive, and shifts that power onto the nurse, who is historically portrayed as a ‘great person’, ‘a saint’, ‘a heroine’ (Dillard‐Wright 2022, p. 33). While framed as compassionate, this stance ultimately reinforces a paternalistic dynamic that centres the nurse's virtue while diminishing the disabled person's agency by assuming the nurse knows what is best for the patient.

Compulsory able‐bodiedness expects a ‘normal’ person to be self‐sufficient, independent, and autonomous. When disabled people do not conform to these ideals, the charity models them as ‘less than’ a person and deserving of pity rather than rights, which reinforces the social hierarchy of able‐bodiedness. Nurses, who are socialised within ableist systems, may unconsciously enact practices that treat disabled patients as dependent and powerless, even when these patients have strong capacities for self‐advocacy and expertise about their own bodies. This ideology maintains able‐bodied supremacy, granting the abled‐bodied person moral superiority for helping a disabled person without challenging their own privilege or confronting ableism, the systems that keep disabled people disempowered in the first place.

Criptiquing the charity model exposes how charity‐oriented practices ultimately serve the identity and power of the [nondisabled] nurse, rather than the liberation or empowerment of disabled people (Boyles et al. 2008). It does so by framing disability as a personal tragedy, rather than a complex interaction of social, political, and environmental factors that limit a disabled person. In doing so, it prevents systemic changes by framing care as a charitable intervention. This protects the professional status of nurses as moral agents (Dillard‐Wright 2023) and distracts from oppressive systems that cause disability in the first place. From a Crip perspective, this is profoundly unethical, as it denies disabled people their rightful place as partners in their own care and contributors to knowledge about their lives (McRuer 2006; Lakshmi Piepzna‐Samarasinha 2018). Rejecting the charity model involves emphasising collective responsibility for removing barriers that disabled people face, rather than pitying them. Nurses can shift towards practices that amplify disabled voices, foster autonomy, and challenge the structures that produce disability in the first place. In doing so, nursing can resist the pull of benevolent paternalism and instead build relationships grounded in solidarity, respect, and shared power.

### 3.3 Criptique of the Medical Model

The medical model of disability has long dominated nursing education and practice (Scullion 2010). Under this paradigm, disability is understood primarily as an individual pathology—a biological defect, impairment, or functional limitation that must be diagnosed, treated, and cured if possible. Nursing curricula and clinical practices grounded in the medical model often emphasise assessment, intervention, and rehabilitation aimed at restoring the disabled person to the ‘mythic norm’ (Lorde 1984, p. 116) of able‐bodiedness. In a system shaped by compulsory able‐bodiedness, the mythic norm is a state of perfection, normalcy, and, most importantly, able‐bodiedness. By engendering the compulsory able‐bodiedness in practice, nurses ‘repeatedly demand that people with disabilities embody for others an affirmative answer to the unspoken question, “Yes, but in the end, wouldn't you rather be more like me?”’ (McRuer 2006, p. 9). Yet, the mythic norm remains an impossibility; humans *do* get sick, develop chronic conditions, have impaired function across the lifespan, and seek nursing care. By choosing to orient towards the social model's rejection of the able‐bodied mythic norm, nursing could instead assert that the able/disabled binary is unnatural (McRuer 2006, p. 37), and there is no perfect human state. Shifting towards the crip paradigm moves nursing education from ‘competence to fix’ to ‘competence to collaborate and adapt’. This transformative shift benefits all individuals who live with varying realities of ability. It also utilises nursing expertise in a more collaborative and less authoritative manner, which fosters relationships with all people seeking to facilitate healing and palliate suffering.

In nursing education, the medical model can train students to view disability through a lens of deficit and dysfunction, rather than as an aspect of diversity. It leaves little space to explore how social, economic, or environmental barriers produce and sustain disablement. As a result, nurses may myopically focus on eliminating impairment, increasing independence while overlooking broader forces of oppression, such as inaccessible infrastructure, ableist policies, or discriminatory attitudes. Moreover, the medical model also fails to meaningfully engage disabled people as partners in care because it portrays them as being *unable*. If disability is treated as only a defect to be managed, then the disabled person becomes a passive recipient of etic expertise rather than an emic collaborator. This erodes opportunities for shared decision‐making and silences the disabled person's lived experience and critical knowledge about their own body and community.

However, ableism in nursing care is not always so explicit. The way student nurses are taught about disability and the role of the [able‐bodied] nurse when caring for the disabled is dogmatic: disability is something we must ‘fix’ and ‘return to normal’. Nurses are oriented towards this rehabilitative logic as a product of their educational roots coming from the medical model of disability. While rehabilitative logic can be helpful for people who will recover from their surgery or acute condition, it is ultimately limiting of nursing interventions if not thoroughly integrated with a crip understanding of disability and well‐being. As McRuer (2006, p. 129) suggests, within a system of compulsory able‐bodiedness, ‘rehabilitation that makes disability disappear (or that promises to do so) is so… preferable to the degradation of living with a disability out in the open’. By medicalizing bodies, medicine and nursing own the ‘problem’ of disability by conceptualising it as a failure of the individual body. This sends a clear message: disability and disabled people should not exist, and so humans should work to erase disability for the betterment of the disabled. This naturalises the assumption that all disabled people desire to be not‐disabled, rather than asking disabled people what they want for themselves. Despite nurses frequently encountering disabled, chronically ill, psychologically stressed, and otherwise ill individuals, the discipline is alarmingly silent in addressing this well‐established issue (Scullion 2010; Boyles et al. 2008).

In its continual use of the medical model's compulsory able‐bodiedness, nurses ‘serve not only their clientele but also themselves, and they are actively involved in perpetuating and expanding their activities’ (Lane 1995, p. 174). Nurses play more roles in growing hospital corporations, ranging from direct patient care to the C‐suite. Nurses have the respect and ethos of Americans as they are famously reputed to be the most trustworthy profession for 22 consecutive years (Brenan and Jones 2024), and that comes with the paternalistic power the medical model grants to experts. Practicing within a crip paradigm would require nurses to relinquish the power they acquired by positioning themselves as the experts of disability, thereby shifting power from themselves back onto the disabled individual. The individual, now centred as the expert on their disability, defines barriers they face when performing a task, and the newly de‐centred nurse can use their expertise to partner with the patient in addressing these barriers. This better reflects the values and goals of the discipline.

### 3.4 The Interplay Between Models

The medical model is so hegemonic that many nurses (and the larger population) are often unknowingly complicit in compulsory able‐bodiedness, having been trained in a rehabilitative logic and working within institutions that seek to (re)produce the able body by medicalizing non‐normative bodies (McRuer 2006). But what does that mean for people whose bodies are permanently disabled and unable to rehabilitate? The moral, charity, and medical models together enforce the stereotype that disabled people are burdens on their families and society or are unable to live successful and meaningful lives. For example, in April 2025, the US Health and Human Services Secretary Robert F. Kennedy Jr. claimed that autism is caused by something [vaccines] the parents are doing to their children, destroys families, is ‘an individual tragedy’, and that autistic children will never play sports, hold jobs, pay taxes, or go on dates (Jacobson 2025; PBS NewsHour 2025). For these reasons, he advocates for an ‘end’ [cure] to autism—a sentiment unshared by autistic people (Natri 2021). Here, Kennedy demonstrates the moral model by suggesting that children are autistic because of the parents’ actions [vaccination], the charity model by depicting autistic children as pitiable tragedies who we have to fix for their betterment, and the medical model by presenting autism as a pathology to cure rather than an example of neurodiversity.

These statements demonstrate the central problem of moral, charity, and medical models of disability, which justify eugenics by rationalising compulsory able‐bodiedness as the clear choice for all humans. By presenting disability as a deficit—be it of moral, financial, or physiological failings—and failing to acknowledge how social, environmental, and political factors influence the experience of disability, the moral, charity, and medical models prime nurses to see cure and elimination of neurodiversity as the obvious solution to a presumed, nonexistent problem. This falsifiable assumption suggests that the individual can regain able‐bodiedness (a neurotypical state, in this example) once more if they follow the nurse's instructions closely enough, take their prescribed treatments as instructed, and *work* to be able‐bodied again. This means that people who fail to rehabilitate to ‘normal’ are not trying hard enough, following instructions closely enough, and are disabled because they are choosing to be, thus justifying their exclusion from an ability‐centred society. This has profound moral, legal, and professional implications for the nursing discipline, which continues to use these three models of disability in its foundational education programmes.

## 4 Reintroducing the Social Model Through a Crip Lens

Crip Theory advocates for more than mere inclusion of disabled people into larger society, wherein they're folded in and forgotten, and instead advocates for a reimagined society that values non‐normative bodies, minds, identities, epistemologies (cripistemologies), and ways of being. The first step in this process is to reconceptualize disability; is it a flaw of the individual's body or mind? Or is it socially constructed? Crips orient away from the moral, charity, and medical models and towards the social model of disability. The social model views disability as a result of social, political, historical, relational, and cultural conditions that create inaccessibility (Oliver 1983). This recognises disabilities as differences in the appearance or function of the body or mind that are not inherently limiting. Instead, disability arises when the tasks are inaccessible, or someone's beliefs about disability limit a disabled person. For example, the social model proposes that the disabling mechanism for a wheelchair user trying to navigate a building that only has stairs is not a dysfunction of their legs, but rather a lack of accessible infrastructure, such as elevators and ramps, with which they would be able to navigate the building without issue. Even the language used to describe a wheelchair user (i.e., ‘wheelchair bound’ or ‘confined to a wheelchair’) reflects able‐bodied assumptions that reflect the model of disability the speaker subscribes to. Wheelchair users tend to emphasise the liberatory nature of wheelchairs as accessibility tools, subverting the able‐bodied assumption that wheelchairs are something that some disabled people are confined or bound to.

The social model of disability contrasts with the medical model by recognising the unaccommodating nature of society and advocates for accessible conditions (Oliver 1983), rather than placing the onus of accessibility on the individual. This difference stems from beliefs about healing. While compulsory able‐bodiedness has led nurses to the misguided belief that ‘you're either sick or well, fixed or broken, and that nobody would want to be in a disabled or sick or mad body mind’ (Lakshmi Piepzna‐Samarasinha 2018, p. 55), most disabled people are not burdened by alterations in their body, but rather by barriers to accommodations and access. By only seeing what people cannot do, nurses can become myopic and overlook the individual's strengths, preferences, and potential, ultimately reducing a whole person to a diagnosis and compromising the quality of care and connection based on unfounded preconceptions. By focusing on deficits, the nurse may incorrectly identify the cause of suffering based on their assumptions about disability and consequently decentre the patient in their own care, which can be a source of suffering in and of itself. This directly contrasts with nursing's emphasis on person‐centred care. Rather, a focus on strengths, preferences, and potential enables the patient to recenter themselves as the expert in their disability, allowing them to self‐identify the sources of suffering. The nurse can then use their expertise to collaboratively address these issues. This emic approach also honours the lived experience of the disabled person, who likely has developed routines, hacks, or adaptations that increase their access in various aspects of their life, exemplifying the innovative nature of disability in nursing practice.

Nurses directly benefit from the tenets of the moral, charity, and medical models of disability, wherein power is granted to the healthcare provider, given their training and expertise in fixing physiological alterations that contribute to disability (Sepasi et al. 2016). As stated previously, the expertise of nursing care is palliation of suffering and facilitation of healing (American Nurses Association 2021). So when the discipline relies on models of disability that subjugates disabled people, the question becomes ‘what do [nurses] think “healing” is? Do [they] think everyone wants to be able‐bodied and neurotypical, and would choose it if they could?’ (Lakshmi Piepzna‐Samarasinha 2018, p. 50). Lakshmi Piepzna‐Samarasinha explains that ‘healing is dismissed as irrelevant, reserved for folks with money, an individual responsibility, something you do on your own time’ (pp. 50–51). This criptique encourages nurses to question whether their concept of healing aligns with the lived experiences and values of disabled people. If healing is narrowly defined as a return to normalcy or able‐bodiedness, then it risks erasing the legitimacy of disabled lives as whole and worthy in their current form. Nursing, when uncritically aligned with the moral, charity, and medical models, may reinforce ableism by pathologizing difference rather than supporting diverse states of health and wellness. Reimagining healing through a disability justice lens means centering on autonomy, community, access, and self‐defined well‐being, rather than simply striving to fix what is perceived as broken.

The social model has limitations. First, the social model can downplay the reality of chronic illnesses or impairment by failing to acknowledge the interaction of biological and social factors that disabled people experience. For example, even if society were fully accessible to a person with chronic pain, they would still experience impairments in their ability to engage with the world around them as a result of their pain. The social model also underplays the importance of assistive devices, medicines, and procedures that offer liberatory potential to disabled people, such as hard‐of‐hearing people who choose to use hearing aids, or people who use ventilators to breathe. The social model could be interpreted by some to suggest that *any* medical interventions are ableist, when the social model proposes that the *pathologization* of disabled bodies is ableist, not necessarily the need for medical interventions themselves. Indeed, many disabled people are grateful for advances in medical technology that address social barriers to communication, mobility, and quality of life (Bonanno et al. 2025). However, compared with other models of disability, the social model provides the most emancipatory potential for nurses and disabled people at this time by challenging more harmful assumptions present in the moral, charity, and medical models.

## 5 Towards a Reimagined Nursing Practice

Reimagining nursing practice through a crip paradigm requires a fundamental reorientation of how disability is conceptualised, taught, and enacted in care relationships. Rather than seeing disability as a deficit or a problem to be solved, nurses can learn to recognise disability as a dimension of human diversity—a source of knowledge, resistance, and transformative possibility. Resisting compulsory able‐bodiedness offers a powerful vantage point for rethinking how nurses engage with disabled people. By refusing the normative frameworks that define ‘good’ bodies and ‘healthy’ lives, nurses can become more attuned to the creativity, adaptability, and resilience that disabled people embody.

The reimagined practice begins in nursing education. Nursing curricula can shift beyond tokenistic inclusion of disability content, moving instead towards a robust engagement with disability culture, history, and critical perspectives. For example, nurses could incorporate first‐person narratives from disabled scholars, integrate disability studies frameworks, and critically interrogate the profession's own complicity in ableist practices. Clinical education should teach students not just to assess physical needs, but also recognise systemic barriers, social determinants, and the historical marginalisation of disabled communities. A curriculum grounded in these insights would prepare nurses to act not as mere fixers of broken bodies, but as partners in dismantling the structures that oppress disabled people.

In practice, nurses can learn to value disabled people as experts of their own bodies and lives. Rather than adopting paternalistic stances of pity or assuming a singular authority on what constitutes health, nurses could instead collaborate with disabled people to co‐create care plans that honour disabled knowledge and lived experience. This approach positions nurses as facilitators of adaptation, access, and autonomy rather than as agents of normalisation.

A crip‐informed nursing requires a broader commitment to policy and structural change. Nursing organisations should prioritise the recruitment and retention of disabled nurses, recognising that disability itself can enrich nursing with perspectives that challenge taken‐for‐granted assumptions about health, independence, and normalcy. Policies and guidelines can be reexamined through a crip lens to root out implicit ableism, including language that frames disability exclusively in medicalized or negative terms.

Ultimately, this reimagined nursing practice is a call to action: for nurses to embrace disability as a form of critical consciousness that disrupts ableist norms, revealing new possibilities for care. In shifting away from models that moralise, pity, or pathologize disabled people, nursing can transform into a more just and liberatory profession—one that values difference as generative and not limiting, resists compulsory able‐bodiedness, centres the disabled person as the expert of their own body, and affirms people's inherent dignity.

## 6 Conclusion

Traditional disability models in nursing are maintained through compulsory able‐bodiedness, which harms the people nurses serve and nurses themselves. Crip theory, particularly the framework of compulsory able‐bodiedness, provides evidence of these subtle patterns in nursing practice, drawing attention to the importance of emic approaches in nursing scholarship and practice. This enables a radically reimagined nursing that fosters a more just, inclusive, liberatory, and transformative practice.

## Ethics Statement

The author has nothing to report.

## Conflicts of Interest

This project supported by the University of California Irvine's Center for Nursing Philosophy Fellowship stipend.

## Data Availability

Data sharing is not applicable to this article as no datasets were generates or analysed during the current study.
